# Emerging Roles of Dysregulated MicroRNAs in Myasthenia Gravis

**DOI:** 10.3389/fnins.2020.00507

**Published:** 2020-05-21

**Authors:** Lin Wang, Lijuan Zhang

**Affiliations:** ^1^Department of Emergency Medicine, Shengjing Hospital of China Medical University, Shenyang, China; ^2^Department of Obstetrics and Gynecology, Shengjing Hospital of China Medical University, Shenyang, China

**Keywords:** microRNA, myasthenia gravis, autoimmune, neuromuscular disease, interleukin

## Abstract

Myasthenia gravis (MG) is a rare acquired autoimmune neuromuscular disease. Autoantibodies, cellular immunity, complement, and cytokines are involved in the pathogenesis of MG. It is characterized by the dysfunction of neuromuscular junction transmission and skeletal muscle weakness. MicroRNAs (miRNAs) are non-coding small molecule ribonucleic acids that regulate various biological processes (e.g., development, differentiation, and immunity) at the transcriptional and post-transcriptional levels of gene expression. miRNAs play an important regulatory role in the pathogenesis of autoimmune diseases, including MG. In recent studies, the functional mechanisms underlying the role of miRNAs in the pathogenesis of MG have received increasing attention. miRNAs are highly stable and have high specificity in peripheral body fluids. Therefore, the miRNAs in body fluids may represent promising biomarkers for determining the prognosis of MG and the efficacy of treatment. This article reviews the role of miRNAs in the pathogenesis of MG, highlights the potential of miRNAs as new biomarkers for the diagnosis of MG, and deepens our understanding of disease processes.

## Introduction

Myasthenia gravis (MG) is an autoimmune disease that is primarily mediated by antibodies against the acetylcholine receptor (AChR) on the postsynaptic membrane of the neuromuscular junction (NMJ) and muscle-specific tyrosine kinase (MuSK), which leads to postsynaptic membrane transmission dysfunction involving both cellular immunity and complement (Gilhus and Verschuuren, 2015; Gilhus, 2016). The annual incidence of MG is about 0.25 to 2 per 100,000 people, mostly in adults (Vincent et al., 2001; Harris et al., 2019). The onset of MG is insidious, and the course of the disease is prolonged. The clinical characteristics of this disease are partial or complete skeletal muscle weakness that can be aggravated by activity. Symptoms are relieved by rest or treatment with cholinesterase inhibitors (ChEI) (Pohanka, 2019; Souto et al., 2019). In severe cases, the medullary and respiratory muscles may be involved, leading to myasthenia crisis (Roper et al., 2017; Barnett et al., 2019).

An abnormal immune response in the thymus is closely associated with the occurrence and development of MG. About 80% of MG patients have thymic hyperplasia or tumors, and a variety of cellular components that induce and maintain abnormal AChR immune responses are located in the proliferative thymus tissue of MG patients (Niendorf et al., 2005; Cordiglieri et al., 2014). The pathogenic mechanism mediated by AChR antibodies (AChR-Ab) includes: (1) AChR-Ab binding to the AChR, activation of complement, and AChR destruction; (2) blocking of the acetylcholine binding site in the AChR by AChR-Ab; (3) crosslinking of AChR by AChR-Ab to accelerate the rate of AChR degradation (Kohler et al., 2017; Huang et al., 2018). Other related pathogenic antibodies identified in MG include MuSK antibodies (MuSK-Ab), low-density lipoprotein receptor (LDLR)-related protein 4 antibodies (LRP4-Ab), and ryanodine receptor antibodies (RyR-Ab) (Morren and Li, 2018). MuSK is a transmembrane protein located on the postsynaptic membrane that can coexist with AChR. A complex between agrin and MuSK can assemble AChR in the postsynaptic membrane. MuSK-Abs inhibit the agrin-MuSK signal, which decreases the concentration of AChR and alters the stability of the NMJ (Huijbers et al., 2019). LRP4, located in the postsynaptic membrane, activates MuSK by binding to the LRP4 receptor to initiate a signaling cascade that promotes the aggregation of AChR. RyR-Abs do not directly cause muscle weakness but may serve as biomarkers of MG (Mantegazza and Antozzi, 2018; Ohnari et al., 2018; Park et al., 2018).

MicroRNAs (miRNAs) are conserved single-stranded non-coding RNAs that bind target mRNAs to regulate gene expression at the post-transcriptional level through the inhibition of translation or degradation of mRNA (Michlewski and Caceres, 2019). More than 30% of the genes in the human genome are thought to be regulated by miRNAs. These genes function in cell proliferation, maturation, differentiation, apoptosis, and other life processes (e.g., immune regulation) (Carthew and Sontheimer, 2009). In recent years, studies have demonstrated that the pathogenesis of MG is associated with thymus abnormalities and multiple genetic and environmental factors; abnormal miRNA function may also be involved in the pathological process of MG (Bo et al., 2019). For example, miR-146 expression was significantly upregulated in MG patients and accompanied by high TLR4, CD40, and CD80 expression levels in AChR-specific B cells (Lu et al., 2013), miR-15a appeared to be involved in the regulation of inflammatory cytokine expression in MG patients (Liu et al., 2016) and miR-125a-5p in the thymus may negatively regulate Foxp expression, leading to an imbalance in autoimmune regulation and MG pathogenesis (Li et al., 2016).

Circulating miRNAs are highly stable and specific and difficult to degrade. Detection of circulating miRNAs can guide the diagnosis and treatment of diseases and provide information on prognosis (Hajjari et al., 2017; Jin and Xing, 2017). Studies have demonstrated that miR-150-5p and miR-21-5p levels are increased in AChR-Ab + MG patients (Punga et al., 2015), suggesting that these two miRNAs may represent promising serum biomarkers for this subset of MG patients. miRNAs are involved in multiple complex and delicate regulatory mechanisms, making the entire signaling pathway a promising therapeutic agent (miRNA mimics) or target (anti-miRNA) in a variety of pathological processes (Kim et al., 2018). At the same time, the advancement of RNA molecular delivery technology has made miRNA-based treatment programs more feasible (Zhao et al., 2015; Wang et al., 2016).

As a relatively rare chronic autoimmune disease of the nervous system, the pathogenesis of MG has not been fully elucidated. In recent years, a number of studies have revealed that dysregulated miRNAs may mediate the pathogenesis of MG through a variety of pathways. In this review, we discuss the emerging roles of dysregulated miRNAs in the pathogenesis of MG in order to provide new ideas for the early diagnosis and introduce miRNA-based therapeutic approaches.

## Biological Origin and Regulatory Mechanism of miRNAs

miRNAs are small single-stranded RNAs of approximately 22 nucleotides (NTs). Although they do not encode proteins, miRNAs can regulate target gene expression and cell function after transcription via an epigenetic mechanism (Ha and Kim, 2014). These molecules can not only bind the 3′-untranslated region (3′-UTR) but also bind the 5′-UTR of mRNA for post-transcriptional regulation of target genes. Through these interactions, miRNAs can regulate cell proliferation, differentiation, and apoptosis, and perform other regulatory roles at various stages of growth and development of organisms (Ferrante and Conti, 2017).

miRNA is thought to be associated with the mechanisms of immunoregulation and activation, including the T-cell response, inflammatory reaction, and expression of inflammatory factors. The study of miRNAs specific to MG may provide valuable information for the pathogenesis and treatment of this disease (Josefowicz et al., 2012; Li et al., 2016; Colamatteo et al., 2019). Dysregulation of miRNA expression and its role in disease can occur as follows: (1) downregulation or deletion of miRNA expression – gene mutation or downregulated or abnormal transcription; (2) miRNA overexpression – overexpression due to gene amplification or mutation inhibits the production of target proteins; (3) Mutation in the 3′-UTR of mRNA affects the miRNA binding site; (4) Mutation in the UTR of mRNA forms a new miRNA binding site, which inhibits the function of affected genes (Quinlan et al., 2017; Fu et al., 2019; Patel et al., 2019).

## The Role of miRNAs in MG Pathogenesis

Several studies using next-generation gene sequencing and high-throughput techniques identified differential expression of miRNAs in the thymus of MG patients. Thymic hyperplasia with ectopic germinal center (GC) is a characteristic pathological change found in early MG (Zuckerman et al., 2010). Sengupta et al. (2018) identified 38 differentially expressed miRNAs in GC-positive patients, two downregulated miRNAs (miR-139-5p and miR-452-5p) were identified by quantitative real-time PCR, and validated a negative regulatory relationship with Regulator of G protein Signaling 13 (*RGS13*), suggesting their involvement in support of GC formation in the thymus. Cron et al. (2018) identified 61 differentially expressed miRNAs (24 up- and 37 downregulated) in a study involving 20 MG patients and five controls. Among them, miR-7-5p and miR-125a-5p were confirmed by qRT-PCR. miR-7 was significantly downregulated and inversely correlated with *CCL21* mRNA expression, while miR-125a was upregulated and associated with *FOXPS* expression (Cron et al., 2018).

**FIGURE 1:**
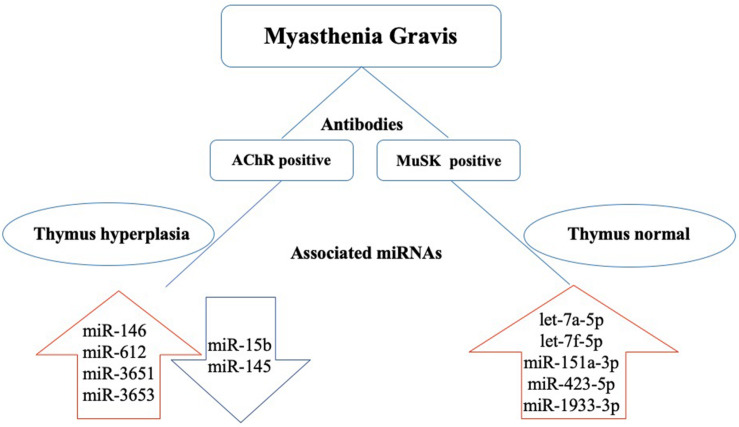
Dysregulated miRNAs in the pathogenesis of MG based on antibody subtype. In acetylcholine receptor antibody seropositive (AChR+) MG, thymus hyperplasia is often found in young patients. miR-146, miR-612, miR-3651, and miR-3653 are upregulated, and miR-15b and miR-145 are downregulated. In muscle-specific tyrosine kinase antibody seropositive (MuSK+) MG, the thymus glands are relatively normal, and let-7a-5p, let-7f-5p, miR-151a-3p, miR-423-5p, and miR-1933-3p are upregulated.

*CXCL13* is a target for the miRNA miR-548k (Li et al., 2018). Dysregulation of miR-548k has been observed in the thymus hyperplasia of MG patients. Thus, dysregulated miR-548k may contribute to MG pathogenesis by regulating CXCL13 mRNA levels (Li et al., 2018). miR-653 was reported decreased in thymocyte and had a negative regulation with tripartite motif 9 (TRIM9), Cao et al. (2019) suggested miR-653 impair proliferation and promote apoptosis of thymocytes of MG mice by suppressing TRIM9. Current studies on the potential role of dysregulated miRNAs in MG pathogenesis are focused on their effect on different MG antibodies and various cytokines secreted by immune cells in this disease. In the next sections, we will discuss the different pathological roles of miRNAs in MG and highlight the potential clinical use of miRNAs as biomarkers or therapeutic targets (Table 1 and Figure 1).

**TABLE 1:** Summary of altered microRNAs (miRNAs) and their targeted cytokines in Myasthenia Gravis (MG).

MiRNAs	Immune cells type	Regulation	Target interleukin	Roles	References
miR-181a	Th1 cell	Down-regulated	IL-2↑	Modulate the activation of CD4+ T cells, expression of transcription factors related to Th1 and Th17 cells.	Liu et al. (2019)
miR-20b	Th1 cell	Down-regulated	IL-8↑, IL-25↑	Inhibit the expression of inflammatory cytokines.	Chunjie et al. (2015)
miR-320a	Th1 cell	Down-regulated	IL-2↑	miR-320a can regulate COX-2 expression through ERK/NF-κB pathways	Cheng et al. (2013)
miR-15b	Th1 cell	Down-regulated	IL-15↑	Regulates IL-15 expression by directly targeting its 3′-UTR	Shi et al. (2015)
let-7c	Th2 cell	Down-regulated	IL-10↑	Regulates IL-10 expression by directly targeting its 3′-UTR	Jiang et al. (2012)
miR-181c	Th17 cell	Down-regulated	IL-7↑, IL-17↑	Negatively regulate immune cell activation	Zhang et al. (2016)
miR-15a	Th17 cell	Down-regulated	IL-17↑, IFN-γ↑	Modulate CXCL10 to increase the expression of cytokines	Liu et al. (2016)
miR-145	B cells	Up-regulated	CD28↓	Play an important role in antigen specific T cells activation	Wang et al. (2013)
miR-146	B cells	Down-regulated	CD40↑, CD80↑	Modulate differentiation and function of cells in innate as well as adaptive immunity	Lu et al. (2013)

## The Role of miRNAs in MG-Related Antibodies

The AChR is present on the surface of muscle cells and concentrated in the synapses between nerve and muscle cells (Bruhova and Auerbach, 2017). Antibodies against the AChR of the postsynaptic NMJ were detected in approximately 80% of MG patients (Cavalcante et al., 2012; Tuzun et al., 2012). Nogales-Gadea et al. (2014) evaluated the levels of miRNAs in the serum of three subgroups of 15 AChR-Ab-positive MG patients (early-onset MG, late-onset MG, and thymoma). They identified 32 differentially expressed miRNAs. miR-15b was expressed at low levels in all three groups, whereas miR-122, miR-140-3p, miR-185, miR-192, and miR-20b were expressed at different levels in early- and late-onset MG compared to the controls (Nogales-Gadea et al., 2014). In a separate study of 19 AChR-positive early-onset MG (AChR-EOMG) patients and 12 controls, Barzago et al. (2016) demonstrated that miR-612, miR-3651, and miR-3653 were upregulated in the peripheral blood mononuclear cells (PBMCs) of these AChR-EOMG patients, suggesting that these dysregulated miRNAs may be involved in the pathogenesis of AChR-EOMG (Barzago et al., 2016). miR-146 is another dysregulated miRNA found in the PBMCs from MG patients with AChR-Ab-positive disease (REF). miR-146 expression was significantly upregulated in MG patients and accompanied by high TLR4, CD40, and CD80 expression levels in AChR-specific B cells (Lu et al., 2013). Based on these results, the authors suggest that the dysregulation of miR-146 could be involved in MG pathogenesis through the regulation of AChR-Ab-specific B cells (Lu et al., 2013). miR-145 was downregulated in the PBMCs from MG patients and CD4+ CD25- T cells from experimental autoimmune MG (EAMG) rats (Wang et al., 2013). CD28 is a target of miR-145 and its levels can be reduced by the upregulation of miR-145 expression. In addition, overexpression of miR-145 can suppress the expression of NFATc1 in AChR-Ab-specific CD4+ T cells and decrease the severity of MG by reducing IL-17 production (Wang et al., 2013).

MuSK is involved in the induction of AChR aggregation during the development of the NMJ, which is crucial for the formation of neuromuscular synapses. MuSK plays an important role in maintaining the homeostasis of the postsynaptic membrane after the NMJ matures. It is speculated that MuSK-Ab cause a decrease in the AChR levels in the postsynaptic membrane by altering MuSK function (Hoch et al., 2001). Previous studies showed that MuSK-Abs are found in 1 to 10% of MG patients and more common in European and American female MG patients. MuSK-Ab-positive MG patients are prone to facial, bulbar, and respiratory muscle weakness that is often accompanied by muscle atrophy. MuSK-Ab-positive MG usually presents with more severe clinical symptoms and is less sensitive to treatment compared to AChR antibody-positive MG (Guptill et al., 2011; Chang et al., 2014; Gilhus and Verschuuren, 2015). Although the incidence of MuSK-Ab-positive MG is relatively low, there have still been a few studies investigating miRNA dysregulation in this type of MG. Bogatikov et al. (2019) identified 13 dysregulated miRNAs (11 upregulated and 2 downregulated) in the omohyoid muscle of mice with MuSK-Ab-positive EAMG. Among these differentially expressed miRNAs, miR-1930-5p and miR-1933-3p had a greater effect on the expression of target genes than other miRNAs, including Impa1 (inositol monophosphatase I) and Mrpl27 (mitochondrial ribosomal protein L27). In addition to these two miRNAs, let-7a-5p, let-7f-5p, mir-151a-3p, and mir-423-5p are specifically elevated in MuSK-Ab-positive MG patients (Punga et al., 2016).

LRP4 is a member of the LDLR family and is the receptor for proteins that are critical for MuSK activation, AChR aggregation, and NMJ formation (Barik et al., 2014). LRP4 antibodies (LRP4-Abs) are detected in 2 to 45% of patients with double seronegative MG (Sieb, 2014). Shen et al. (2013) first reported LRP4-Abs in the blood of patients with MG. Injection of LRP4-Abs into healthy mice caused typical MG symptoms, validating the involvement of LRP4-Abs in the pathogenesis of MG (Shen et al., 2013). Few studies have focused on the relationship between dysregulated miRNAs and LRP4-Abs. LRP4 is a target for miR-490-3p (Xie et al., 2019). It can also be a target of miR-196a, miR-1232, and miR-1544; however, there are no studies on the relationship between these miRNAs and LRP4 in MG (Li et al., 2015; Karakatsani et al., 2017).

Agrin is a basement membrane protein that includes two isoforms, neuronal agrin (N-agrin) and muscular agrin (M-agrin). N-agrin binds to LRP4-Ab, activates MuSK-Ab, induces the aggregation of AChR-Abs, and plays an important role in NMJ structure formation and maintenance (Koneczny and Herbst, 2019). Titin antibodies are raised against the major immunogenic region (MIR) at the A/I band junction. The effects of the Titin antibodies are closely related to MG that is complicated with thymoma, and these antibodies are important for the diagnosis of this subtype of MG (Szczudlik et al., 2014). At present, the relationship between miRNAs and these two antibodies remains to be studied.

## miRNAs and MG-Related Immune Cells and Cytokines

CD4+ T cells, also known as helper T cells (Th), usually include three subpopulations of cells (Th1, Th2, and Th3). In MG patients, Th1 and Th2 cells can act on AChR-Ab-specific B cells by secreting cytokines to enhance the humoral immune response, while Th3 cells mainly exert inhibitory effects by secreting transforming growth factor-β (TGF-β) (Pianta et al., 2015). Mature B cells can be stimulated by antigen by peripheral lymphatic cells. With the help of Th and antigen-presenting cells and the cytokines produced by them, B cells can be activated to proliferate and differentiate into plasma cells that synthesize and secrete antibodies. For example, Cron et al. (2019) demonstrated that miR-150 expression was downregulated in the PBMCs from MG patients, especially in CD4+ T cells. They also observed upregulated miR-150 expression in the thymus, which was associated with the involvement of thymic B cells in MG (Cron et al., 2019).

Th1 cell-specific cytokines include interleukin-2 (IL-2), IL-12, interferon-γ (IFN-γ), and tumor necrosis factor-α (TNF-α), which initiate cellular immune responses (Buonocore et al., 2019). IL-2 is a cytokine that plays an immune-enhancing role by inducing the proliferation of Th1 cells and producing IFN-γ to promote B-cell proliferation and the secretion of antibodies (Buonocore et al., 2019). miR-181a is downregulated in PBMCs from MG patients and related to the levels of AChR antibody. miR-181a is involved in the modulation of CD4+ T-cell activation and associated with the expression of transcription factors related to Th1 and Th17. Liu et al. (2019) found that IL-2 is regulated by miR-181a. There is a negative correlation between the levels of miR-181a and IL-2, suggesting that miR-181a is involved in the pathogenesis of MG through the modulation of IL-2 expression (Liu et al., 2019). IL-8 is a chemotactic cytokine that promotes inflammatory cell chemotaxis and induces cell proliferation. IL-8 is secreted by Th1 cells, IL-8 is secreted by Th1 cells and mediate cytotoxic and local inflammation-related immune response-assisted antibody production. Its main biological functions are to activate neutrophils and promote neutrophil lysosomal enzyme activity and phagocytosis (Meniailo et al., 2018). IL-25 is a new member of the IL-17 family. It plays an important regulatory role in type 2 immune reaction. Chunjie et al. (2015) found that the levels of IL-8 and IL-25 are increased in the serum from MG patients. They also found that the expression of miR-20b was downregulated in these MG patients. There was a direct negative relationship between miR-20b and IL-8 and IL-25. The down-regulated miR-20b expression could altered the serum IL-8 and IL-25 levels, exacerbating cytotoxic and local inflammation-related immune response-assisted antibody production (Chunjie et al., 2015). Cheng et al. (2013) found that miR-320a is downregulated in MG patients. Their data suggest that miR-320a is involved in regulating the extracellular-regulated protein kinase (ERK)/nuclear factor kappa-B (NF-κB) pathway, which in turn induces the expression of cyclooxygenase 2 (COX-2) and promotes the expression of IL-2 and IFN-γ (Cheng et al., 2013). Together, these effects may contribute to MG pathogenesis. IL-15 is produced by a variety of cells (e.g., activated monocytes and macrophages, epidermal cells, and fibroblasts). IL-15 induces B-cell proliferation and differentiation, stimulates T-cell and NK-cell proliferation, and also stimulates NK cells to produce IFN-γ (Hromadnikova et al., 2016). The expression of IL-15 is significantly higher in patients with eye MG, limb MG, or thymomas compared to healthy controls. miR-15b is downregulated in MG patients. Shi et al. (2015) suggested that downregulated miR-15b targets IL-15 and negatively regulates its expression, to induce B-, T-, and NK-cell proliferation and differentiation, and involves in MG progression.

Th2 cells mainly secrete IL-4, IL-6, IL-9, and IL-10, which promote B-cell proliferation, differentiation, and antibody formation and initiate humoral immunity. IL-10 levels are significantly higher in MG patients compared to controls and correlate with the clinical severity of MG (Yeh et al., 2009). Jiang et al. (2012) found that the let-7 family of miRNAs was downregulated in the PBMCs from MG patients. IL-10 is a target of let-7c, and its expression levels are inversely correlated with that of let-7c (Jiang et al., 2012). Thus, dysregulated let-7c expression may contribute to the initiation and progression of MG through its regulation of IL-10 (Jiang et al., 2012).

The relative lack of Treg cells is a common feature of human autoimmune diseases. The thymus produces most of the Treg cells in the human body. Forkhead box P3 (Foxp3) is a key transcription regulator of the immunosuppressive function of Treg cells. Downregulation of Foxp3 expression can lead to the loss of Treg cell function (Kumar et al., 2019). Li et al. (2016) found that miR-125a-5p is highly expressed in the thymus of MG patients with thymomas, and Foxp is a target gene for miR-125a-5p. In MG patients with thymoma, miR-125a-5p in the thymus may negatively regulate Foxp expression, leading to an imbalance in autoimmune regulation and MG pathogenesis (Li et al., 2016).

In addition to CD4+ T cells, Th17 cells represent another cell subpopulation involved in the pathogenesis of MG. IL-7 is a multipotent cytokine that is required for the growth of T and B cells and resistance to apoptosis. It can maintain the stability of the immune system and promote the proliferation and differentiation of CD4+ and CD8+ T cell subsets in the thymus (Vanhanen et al., 2018). As an important cytokine secreted by Th17 cells, IL-17 can induce the expression of various chemokines and cytokines, participate in immune cell and inflammatory responses, and play an important role in the pathogenesis of autoimmune diseases (Khan and Ghazanfar, 2018). Increased IL-7 and IL-17 serum levels have been found in MG patients (REF). Zhang et al. (2016) demonstrated that miR-181c is downregulated in the PBMCs from MG patients and is negatively correlated with the levels of both IL-7 and IL-17. Increased expression of miR-181c caused a decrease in the IL-7 and IL-17 levels released from cultured PBMCs, relieving immune cell-mediated inflammation (Zhang et al., 2016). miR-15a appears to be involved in the regulation of inflammatory cytokine expression in MG patients (Liu et al., 2016). Interferon-gamma-inducible protein 10 (CXCL10) is a direct target gene for miR-15a in MG. The downregulation of miR-15a expression leads to the upregulation of CXCL10 expression and subsequent increases in the expression of IL-17 and IFN-γ (Liu et al., 2016).

B cells originate from the bone marrow and enter all peripheral lymphoid organs of the human body as part of the humoral immune system. Dysregulation of miRNAs can lead to changes in the number of B cells, functional defects, or overactivation. It also causes the disorder of complement and cell surface molecules, resulting in the release of a large number of autoantibodies that attack the NMJ and participate in the pathogenesis of MG. CD28 is a target for miR-145 and can be reduced by the upregulation of miR-145 expression in EAMG rats (Wang et al., 2013). CD40 and CD80 are highly expressed in AChR-specific B cells and are regulated by dysregulated miR-146 expression, suggesting miR-146 may participate importantly in the regulation of AchR specific B cells and involve in the pathogenesis of MG (Lu et al., 2013).

## miRNAs as Biomarkers of MG

Serum AChR, Titin, RyR, and MuSK antibodies have all been implicated in the pathogenesis of MG. Changes in their levels are helpful for the diagnosis of MG. However, the positive rate for the AChR antibodies in MG patients without thymoma is 36.8% (Meriggioli and Sanders, 2012). The positive rate of serum Titin antibodies in MG patients is 27% (Szczudlik et al., 2014). Although this positive rate may be helpful for early diagnosis, changes in Titin antibody levels fail to reflect the progression of the disease or its response to treatment (Szczudlik et al., 2014).

Circulating miRNAs are stable molecules in the blood that can be detected by non-invasive, highly sensitive methods. Previous studies demonstrated that many miRNAs could be used as biomarkers for disease diagnosis, including in MG. Several studies demonstrated that miR-150-5p and miR-21-5p levels are increased in AChR-Ab + MG patients (Punga et al., 2015), suggesting that these two miRNAs may represent promising serum biomarkers for this subset of MG patients. The levels of miR-150-5p may also correlate with MG severity. In contrast, miR-20b is downregulated in AChR+ MG patients compared to healthy controls (Chunjie et al., 2015). The levels of the let-7 family of miRNAs are elevated in MuSK-Ab + MG patients (Punga et al., 2016; Punga and Punga, 2018). Plasma miR-210-3p and miR-324-3p levels are decreased in MuSK+ MG patients compared to healthy controls (Sabre et al., 2018a).

In addition to their diagnostic value, miRNAs may also be predictive of the prognosis of MG patients. It was reported that the miR-30e-5p levels are elevated in ocular MG patients and had high sensitivity in differentiating ocular MG and secondary generalized MG of late-onset ocular MG patients (Sabre et al., 2019). Sabre et al. (2018b) found that the levels of miR-21-5p and miR-150-5p were higher in generalized late-onset MG (LOMG) compared to ocular LOMG and positively correlated with the age of the MG patients. miR-21-5p, miR-30e-5p, and miR-150-5p levels were decreased concurrently with clinical improvement after immunosuppression therapy, and their levels were positively correlated with the clinical MG composite score (Sabre et al., 2018b). miR-150-5p decreased 24 months after thymectomy, providing new evidence and further support for miR-150-5p as a biomarker for MG (Molin et al., 2018). Cavalcante et al. (2019) identified miR-323b-3p, miR-409-3p, and miR-485-3p as predictive biomarkers for the responsiveness of MG patients to immunosuppressive drugs. miR-181d-5p, miR-323b-3p, miR-340-3p miR-409-3p, and miR-485-3p have also been validated as drug efficacy biomarkers (Cavalcante et al., 2019).

## miRNA-Based Therapeutic Approaches for MG

Biological drugs, such as immunosuppressants, have broad clinical prospects in the treatment of MG. Immunosuppressants currently used include monoclonal antibodies (e.g., eculizumab, rituximab, and belimumab) and the immunoglobulin regulator Efgartigimod (Mantegazza et al., 2018).

miRNAs are involved in MG immune disorders through a variety of mechanisms. Modulating the expression of pathogenic genes or inhibiting the activation of immune cells and the release of inflammatory cytokines may represent a novel strategy to treat MG. For example, overexpression of miR-146a in exosomes from dendritic cells was recently found to have antigen-specific suppressive effects in EAMG (Yin et al., 2017). miR-155 is a better-characterized miRNA that is involved in inflammation and the immune reactions observed in MG. Wang et al. (2014) have proposed that the silencing of miR-155 could reduce the translocation of nuclear factor (NF)-κB into the nucleus, making it a promising target for the treatment of MG. However, an additional challenge exists for the use of miRNA mimics or antagomir in the treatment of MG due to miRNA degradation. Moreover, although virus vector-mediated, liposome-based, and gold nanoparticle delivery methods may be useful, further studies on miRNA delivery are also needed.

## Conclusion

In conclusion, MG is an autoimmune disease of the nervous system. Its immunological pathogenesis has attracted much attention, particularly the production of autoantibodies, impairment of immune cell function, and the imbalance of cytokines. As important posttranscriptional regulatory factors, miRNAs play a key role in the development of MG. Studying the role of miRNAs in the immunomodulation reaction of MG is of great importance to the clarification of the pathophysiological mechanism underlying this disease. In addition, circulating miRNAs are promising biomarkers for diagnosing and evaluating disease severity, and the development of miRNA-based treatment may also provide a new therapeutic strategy against MG.

## Author Contributions

This manuscript was primarily written by LW. The figure was produced by LW and LZ. LZ contributed to the editing of this review. Both authors read and approved the final manuscript.

## Conflict of Interest

The authors declare that the research was conducted in the absence of any commercial or financial relationships that could be construed as a potential conflict of interest.
